# The Differential Impact of Clear Aligners and Fixed Orthodontic Appliances on Periodontal Health: A Systematic Review

**DOI:** 10.3390/children12020138

**Published:** 2025-01-26

**Authors:** Gianna Dipalma, Alessio Danilo Inchingolo, Arianna Fiore, Liviana Balestriere, Paola Nardelli, Lucia Casamassima, Daniela Di Venere, Andrea Palermo, Francesco Inchingolo, Angelo Michele Inchingolo

**Affiliations:** 1 Department of Interdisciplinary Medicine, University of Bari “Aldo Moro”, 70121 Bari, Italy; giannadipalma@tiscali.it (G.D.); ad.inchingolo@libero.it (A.D.I.); arianna.fiore@uniba.it (A.F.); liviana.balestriere@uniba.it (L.B.); paola.nardelli@uniba.it (P.N.); lucia.casamassima@uniba.it (L.C.); daniela.divenere@uniba.it (D.D.V.); a.inchingolo3@studenti.uniba.it (A.M.I.); 2 Department of Interdisciplinary Medicine, University of Salento, 73100 Lecce, Italy; andrea.palermo2004@libero.it

**Keywords:** periodontal health, orthodontic treatment, periodontitis

## Abstract

Background/objective: In orthodontic therapy, the periodontal ligament plays a critical role in the bone remodeling process by stimulating osteoblasts in tension zones and promoting bone resorption through osteoclasts in compression zones in response to mechanical stress. These processes are regulated by key cytokines, such as RANKL and IL-1, which are influenced by factors such as patient age and force application. This work evaluates the effectiveness of clear aligners versus traditional braces on periodontal health in patients with periodontitis, following PRISMA guidelines and utilizing specific inclusion and exclusion criteria. Methods: A systematic review of 1664 records was conducted, leading to the inclusion of eight studies that focus on the impact of orthodontic treatments on periodontal health. The review identifies various biases present in the literature. Results: The findings reveal that clear aligners, in contrast to fixed appliances, improve oral hygiene and reduce inflammation, leading to better periodontal outcomes. Fixed appliances, on the other hand, may exacerbate plaque accumulation and inflammation, which can worsen periodontal health. Conclusions: Clear aligners offer advantages over fixed appliances in terms of enhancing periodontal health, improving patient compliance, and providing long-term benefits, particularly in patients with severe periodontitis. The effectiveness of clear aligners is linked to better management of periodontal complications and overall oral hygiene. Treatment decisions should be based on patient-specific criteria to optimize outcomes.

## 1. Introduction

**Figure 1:**
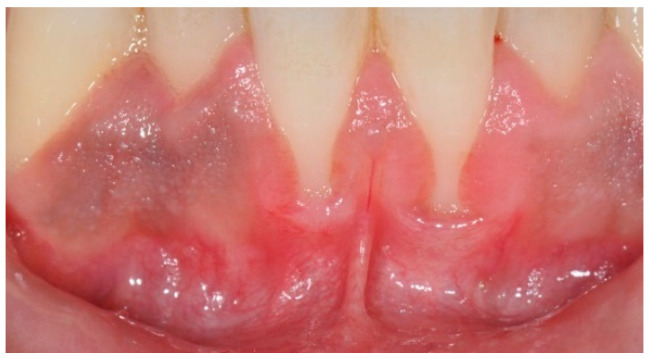
Gingival recession after orthodontic treatment.

The periodontal ligament (PDL) and surrounding tissues undergo a variety of physiological changes because of the mechanical stresses that are applied to teeth during orthodontic therapy [1,2,3]. Between the cementum of the tooth and the alveolar bone, the PDL is a specialized connective tissue that is essential for absorbing and dispersing mechanical stresses during mastication [4]. This ligament goes through major remodeling processes during orthodontic tooth movement, which is essential for correctly relocating teeth [5,6,7,8] (Figure 1).

The PDL experiences areas of tension and compression when orthodontic pressures are applied, which results in different biological reactions [9,10,11]. PDL fibers are compressed, blood supply is decreased, and cellular reactions are triggered in compression zones to cause bone resorption, which facilitates tooth movement [12,13,14]. On the other hand, in tension-affected locations, the PDL fibers are stretched, which encourages osteoblastic activity and bone formation, helping to maintain the tooth in its new position [15,16,17,18].

The PDL’s mechanical stress causes various cellular and molecular alterations [4]. According to studies, the use of mild orthodontic stress increases the expression of important proteins including collagen types I and IV, which are necessary for preserving the PDL’s structure and functionality [19]. An extracellular matrix protein called fibronectin is important in the healing processes that occur after mechanical stress [20]. Simultaneously, during orthodontic treatment, proinflammatory cytokines including prostaglandins and interleukin-1 (IL-1) influence bone remodeling [21,22,23,24,25].

The biological mechanism involved in orthodontic tooth movement is a highly regulated process that involves various cells, tissues, and biochemical mediators [26,27,28,29,30]. The following is a scheme that outlines the key components involved in the PDL and surrounding tissues during orthodontic treatment:

### 1.1. Cells Involved

- Osteoblasts: responsible for bone formation on the tension side of the probing depth (PD) [28,29,30].
- Osteoclasts: cells that resorb bone on the pressure side to allow tooth movement [31,32,33,34,35,36,37,38,39,40].
- Fibroblasts: predominant cells in the PDL, involved in the synthesis and remodeling of the extracellular matrix [41,42,43,44].
- Endothelial cells: involved in the vascular changes that occur during the remodeling process [45,46].
- Macrophages: play a role in the removal of necrotic tissue during bone resorption [46].
- Cementoblasts: responsible for the formation and repair of cementum on the root surfaces of teeth [46].
- Osteocytes: mechanosensory cells in the bone, they detect mechanical load and contribute to bone remodeling [20].

### 1.2. Tissues Involved

- PDL: connective tissue between the tooth root and alveolar bone that experiences mechanical stress during orthodontic treatment. It contains collagen fibers, blood vessels, and nerves [4].
- Alveolar bone: the bone that undergoes remodeling—resorption on the pressure side and deposition on the tension side [47,48,49].
- Cementum: the mineralized tissue covering the root of the tooth, which serves as the attachment point for PDL fibers [50,51].

### 1.3. Biochemical Mediators and Pathways

Cytokines:

- IL-1, Interleukin-6 (IL-6), and tumor necrosis factor-alpha (TNF-α): these inflammatory cytokines promote the recruitment and activation of osteoclasts, facilitating bone resorption on the pressure side [52].
- Prostaglandins (PGE2): mediate vasodilation and are critical for osteoclastic activity and bone resorption [53].
- Receptor activator of nuclear factor kappa-B ligand (RANK): stimulates osteoclast formation, leading to bone resorption on the pressure side [54,55,56].

### 1.4. Mechanical Forces and Responses

Pressure side:

- Blood flow is reduced, leading to hypoxia and necrosis (hyalinization). Osteoclasts are recruited to resorb bones, allowing the tooth to move into the space created [57].

Tension side:

- The PDL is stretched and blood flow increases, promoting osteoblastic activity. This results in bone formation to stabilize the teeth in their new position [58,59,60,61,62].

### 1.5. Phases of Tooth Movement

- Initial phase: quick tooth movement due to PDL deformation and extrusion of PDL fluid [57].
- Lag phase: slowed movement as hyalinized tissue is removed by macrophages and osteoclasts [63,64,65].
- Post-lag phase: accelerated movement due to continued bone remodeling and formation [19].

**Figure 2:**
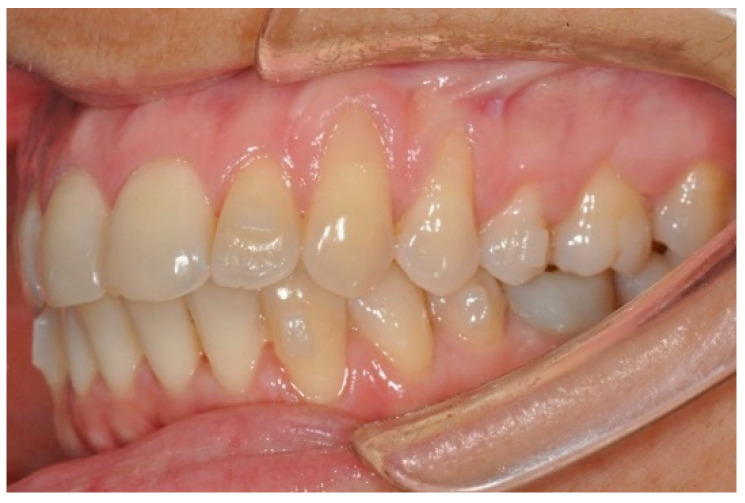
Gingival recession during treatment with aligners.

The strength and duration of the applied pressures affect the PDL remodeling [66]. Overuse of force can have negative consequences, such as root resorption, which is the disintegration of the root structure caused by the recruitment of odontoclasts to resorb mineralized tissues in response to mechanical stress [57]. Furthermore, the reaction to orthodontic forces varies with age. Specifically, younger patients’ teeth tend to move more quickly because of increased PDL metabolic activity, whereas elderly patients’ teeth may move more slowly because of decreased cellular reactivity [67,68,69,70] (Figure 2).

## 2. Materials and Methods

### 2.1. PICO Question

The P(Population) I (Intervention) C (Control) O (Outcome) approach is used to evaluate the effect of an intervention on a specific condition, in this case, the effect of clear aligners and braces on periodontal health.

In patients with periodontitis (P), is the use of clear aligners (I) more effective than fixed orthodontic treatment with traditional braces (C) in improving periodontal health, reducing inflammation, and maintaining long-term periodontal stability (O)?

### 2.2. Protocol and Registration

Our search was performed following the method of Preferred Reporting Items for Systematic Reviews and Meta-Analysis (PRISMA) guidelines and registered in the International Prospective Register of Systematic Review Registry guidelines (PROSPERO ID: 609357).

### 2.3. Search Processing

The electronic databases PubMed, Scopus, and Web of Science were searched to find papers matching our topic dating from 1 January 2014 to 1 September 2024. The Medical Subject Headings (MESH) terms entered in search engines were: “periodontal health” AND “orthodontic treatment” (Table 1).

### 2.4. Inclusion and Exclusion Criteria

The inclusion criteria were the following: (1) English language; (2) any type of observational study, e.g., retrospective cohort, prospective cohort, case-control, cross-sectional, and randomized controlled trials; (3) open access; (4) articles concerning the relationship between orthodontic treatment and periodontal health (5) only adolescents and adults.

The exclusion criteria were the following: (1) other languages except English; (2) reviews and meta-analyses; (3) off-topic articles; (4) in vivo studies; (5) in vitro studies.

### 2.5. Data Processing

The reviewers screened the records according to the inclusion and exclusion criteria. Doubts were resolved by consulting the senior reviewers (F.I.). The selected articles were downloaded into Mendeley.

## 3. Results

### 3.1. Study Selection and Characteristics

A total of 1664 records were identified using the keywords “periodontal health” AND “orthodontic treatment”. When applicable, the automatic filters entered were only in English, only clinical studies, only humans, no reviews, and free full text. The consulted databases were PubMed (1253), Scopus (123), and Web of Science (288).

During the screening phase, the inclusion and exclusion criteria were applied based on the analysis of the title and the abstract and included only studies that focused on the relationship between orthodontic treatment and periodontal health in adolescents and adults.

**Figure 3:**
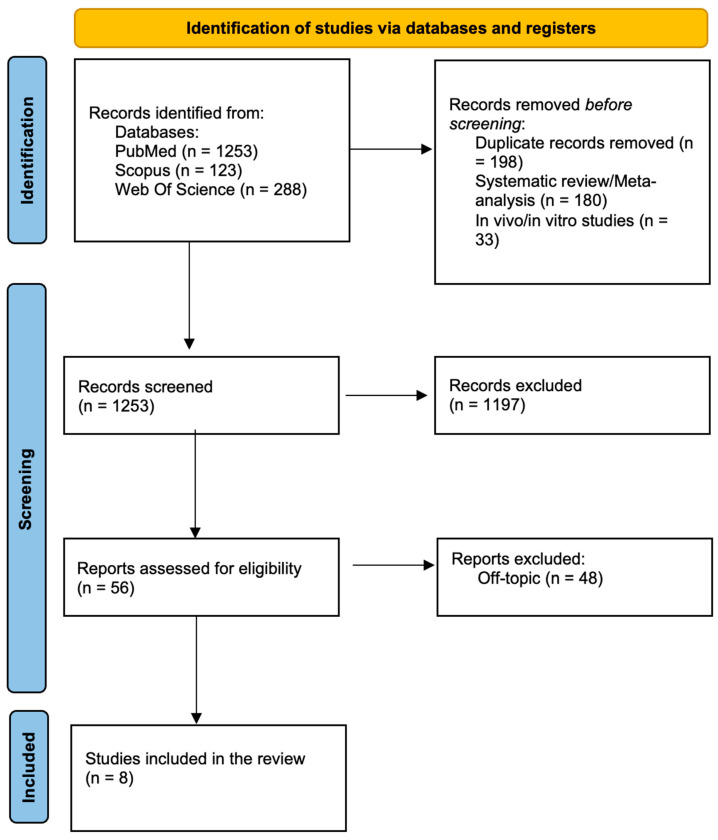
PRISMA flow chart.

After screening, 197 duplicate articles, 180 systematic reviews, and 33 in vivo/in vitro studies were excluded. Then, 1197 articles were excluded by the analysis of title and abstract, leading to 56 records assessed for eligibility. After determining eligibility, eight studies (Table 2) were included in the final analysis. The process is summarized in Figure 3.

### 3.2. Quality Assessment and Risk of Bias of Included Articles

The risk of bias in the included studies is reported in Table 3.

The quality assessment and risk of bias analysis were therefore based on seven critical domains assessing potential threats to validity: confounding bias, measurement of exposure, participant selection, post-exposure interventions, missing data, measurement of outcomes, and selection of reported results. The review by Abdelhafez et al. (2021) [72] had a high risk noted for bias in the confounding and participant selection domains, thus leading to many questions about the accuracy and generalization of the findings. On the other hand, Abbate et al. (2015) [71] found only minimal hazards in most areas, except for some issues with measurement bias. Papers like those by Azaripour et al. (2015) [73] and Hye-Young et al. (2017) [74] gave a mixed risk profile where hazards were lower in certain areas while having substantial biases in other areas, such as exposure and outcome measurement, for instance. Confounding and missing data were the serious biases for Pango Madariaga et al. (2020) [77], biases that might have distorted their findings. The repeated instances of areas with “some concerns” for several studies demonstrate the need for better study designs, despite the admirable methodological rigor in some. If there is to be an improvement in the validity of research findings, ensuring their practical usefulness, these biases must be addressed.

## 4. Discussion

Orthodontic treatment plays a crucial role in achieving optimal dental alignment and improving overall oral health. However, the impact of different orthodontic approaches—particularly fixed appliances and clear aligners—on periodontal health has become an area of significant interest. Understanding how these treatments affect plaque accumulation, gingival inflammation, and periodontal outcomes is essential for both practitioners and patients [79,80,81]. As orthodontics continues to evolve with advancements in technology and treatment methodologies, it is imperative to evaluate the potential benefits and drawbacks of these approaches, especially for populations at higher risk for periodontal issues [82,83,84]. This investigation aims to shed light on the intricate relationship between orthodontic interventions and periodontal health, ultimately guiding informed decision-making for effective treatment outcomes [85,86,87,88,89,90].

### 4.1. Impact on Plaque Accumulation and Inflammation

Several studies, including those by Kumar et al., Abbate et al., and Levrini et al., demonstrate that fixed appliances are linked to increased plaque accumulation and gingival inflammation [91,92,93,94]. The mechanical complexity of brackets and wires creates retention areas that trap bacteria and impede effective cleaning [95,96,97]. Kumar et al. report significant increases in visible plaque and gingival recession among patients undergoing treatment with fixed orthodontic appliances, highlighting the inflammatory consequences these devices impose on gingival tissues [71,75,76,98]. Similarly, Abbate et al. corroborate these findings, noting elevated plaque scores and bleeding on probing (BOP) in adolescents utilizing fixed appliances. Levrini et al. further indicate that fixed appliances contribute to greater biofilm formation and periodontal inflammation in comparison to Invisalign aligners [76,99,100,101,102].

In contrast, clear aligners are associated with more favorable periodontal outcomes. Studies by Levrini et al. and Azaripour et al. reveal that aligners facilitate superior maintenance of periodontal health, primarily due to their removability, which allows for more effective oral hygiene practices. Azaripour et al. also report higher patient satisfaction and fewer negative impacts on dietary habits and self-perception when using aligners [103]. These findings suggest that clear aligners may be more suitable for individuals concerned about both oral health and aesthetics during orthodontic treatment [73,104,105,106,107].

### 4.2. Periodontal Health in Adolescents vs. Adults

Patient age appears to significantly influence the periodontal outcomes observed with various orthodontic treatments. Abbate et al. focus on adolescents, finding that Invisalign aligners promote improved compliance and hygiene in this demographic [1,2,108,109]. This assertion is supported by Ravera et al., who observed that the digital integration of aligner therapy yields more favorable periodontal results in adults with advanced periodontitis. Conversely, Madariaga et al. demonstrated that with appropriate professional hygiene interventions, both fixed and removable devices achieve similar improvements in periodontal health after three months, underscoring the importance of supervised oral hygiene regardless of appliance type [71,77,78].

### 4.3. Impact of Orthodontic Treatment on Gingival and Bone Health

The study conducted by Abdelhafez et al. examines the effects of orthodontic treatment on gingival and bone health in adults [110,111,112,113,114]. Their findings indicate that orthodontic treatment tends to reduce the width of keratinized gingiva and crestal bone levels; however, these alterations remain within clinically acceptable limits. Notably, differences arise between patients who undergo extractions and those who do not, with extraction cases showing further reductions in keratinized gingiva and less dental exposure [115,116,117]. This suggests that orthodontic treatment may induce minor yet manageable alterations in periodontal health that could influence aesthetic outcomes in adults [72,118,119,120].

### 4.4. Influence of Orthodontic History on Periodontal Disease Incidence

Sim et al. provide a unique perspective by analyzing data from a large national survey, linking past orthodontic treatment with a lower incidence of periodontitis. They propose that orthodontics may confer a protective effect against periodontal diseases, potentially due to improved dental alignment and ease of maintenance in treated individuals. This perspective contrasts with other studies focusing on the immediate periodontal challenges posed by orthodontic appliances, suggesting instead a long-term benefit for periodontal health [74,121].

### 4.5. Effectiveness of Aligner Therapy in Severe Periodontitis

Ravera et al. investigated the application of aligner therapy in patients diagnosed with Stage IV periodontitis, demonstrating positive improvements in periodontal health, including reductions in probing pocket depth (PPD) and clinical attachment loss (CAL) [122,123,124]. Their findings support the therapeutic potential of aligners in managing complex periodontal cases, emphasizing the advantages of aligners in minimizing occlusal trauma—a frequent concern for patients with compromised periodontal status. This agrees with findings from other studies, such as those by Azaripour et al. and Levrini et al., indicating that aligners are particularly beneficial for individuals with periodontal concerns, effectively managing both aesthetic and functional outcomes [73,76,125,126].

In conclusion, the collective findings of these studies suggest that clear aligners may be preferable for patients at heightened risk of periodontal complications or those facing hygiene challenges. Aligners facilitate more effective cleaning, reduce inflammation, and enhance patient compliance [127,128,129]. Conversely, while fixed appliances are effective for complex orthodontic corrections, they are more likely to exacerbate plaque accumulation and gingival recession, necessitating stringent hygiene protocols to mitigate their periodontal impacts [130,131,132]. Ultimately, patient-specific factors—such as age, motivation, and periodontal status—should guide the choice between aligners and fixed appliances, ensuring optimal outcomes for periodontal health [133,134,135].

### 4.6. Limitations and Future Directions

While this systematic review provides valuable insights into the impact of orthodontic treatment methods on periodontal health, several limitations warrant consideration [136,137,138]. The heterogeneity among studies in terms of design, sample sizes, follow-up periods, and treatment protocols complicates direct comparisons and limits the generalizability of the findings. Additionally, variations in data collection methods and outcome measures further obscure the ability to draw definitive conclusions regarding the relative efficacy of fixed appliances versus clear aligners. Some studies with small sample sizes raise concerns about potential bias, highlighting the need for larger, well-designed clinical trials to enhance the robustness of the evidence base [139,140,141,142,143,144].

To address these challenges and advance our understanding of orthodontic interventions, standardized protocols and collaborative efforts among researchers are crucial [145,146,147]. Future research should focus on longitudinal studies that track periodontal health outcomes over extended periods, allowing for a comprehensive assessment of the long-term effects of different orthodontic modalities. Exploring the psychosocial impacts of orthodontic treatment on patients’ quality of life could also yield important insights, particularly regarding aesthetic considerations and perceived oral health [148,149,150].

Moreover, the integration of emerging technologies and innovative treatment approaches may revolutionize the field, offering more effective and patient-centered solutions. As we continue to investigate the relationship between orthodontic treatments and periodontal health, sustained collaboration and a commitment to innovation will be essential to unlocking the full potential of these therapeutic strategies and improving patient care [151,152,153].

## 5. Conclusions

In analyzing the outcomes of this systematic review, several key observations emerge regarding the impact of orthodontic treatment methods on periodontal health:

Plaque Accumulation and Gingival Inflammation: Fixed appliances are associated with increased plaque accumulation and gingival inflammation compared to clear aligners, which facilitate better oral hygiene due to their removability.

Patient Compliance: Clear aligners promote higher compliance and improved periodontal outcomes in adolescents as they allow for easier maintenance of oral hygiene practices.

Long-term Periodontal Health: Past orthodontic treatment appears to correlate with a lower incidence of periodontitis, suggesting potential long-term benefits for periodontal health associated with improved dental alignment.

Treatment for Severe Periodontitis: Clear aligners demonstrate positive effects on periodontal health in patients with severe periodontitis, effectively reducing probing pocket depth and clinical attachment loss [154,155,156,157].

In essence, while both fixed appliances and clear aligners achieve effective orthodontic corrections, clear aligners show significant advantages in promoting periodontal health, enhancing patient compliance, and minimizing the risk of complications, positioning them as a preferable option for patients, particularly those at heightened risk for periodontal issues.

## Figures and Tables

**Table 1:** Article screening strategy.

**Article** **Screening Strategy**	KEYWORDS: A: “periodontal health”; B: “orthodontic treatment”
	Boolean Indicators: “A” AND “B”
	Timespan: from 1 January 2014 to 30 September 2024
	Electronic Databases: PubMed, Scopus, and Web of Science

**Table 2:** Analysis of the studies included in the discussion section.

Authors	Type of Study	Patients	Number of Patients Treated with Clear Aligners	Number of Patients Treated with Fixed Appliances	Aim of the Study	Materials and Methods	Conclusions
Abbate G.M. et al., 2015 [71]	Randomized trial	50 adolescents; both sexes	25	25	Evaluated periodontal health over 12 months in two groups (fixed braces vs. Invisalign); measured plaque, bleeding on probing (BOP), PD, and microbiological changes	To assess periodontal health differences in adolescents using fixed braces versus Invisalign	Invisalign showed better periodontal health with reduced plaque and BOP; fixed braces led to increased plaque, bleeding, and higher periodontal indices. Aligners allowed better hygiene compliance, contributing to better periodontal health outcomes.
Abdelhafez R.S. et al., 2021 [72]	Comparative cross-sectional	311 patients: 249 females, 62 males	Not specified	Not specified	Assess the effects of orthodontic treatment on the periodontium and tissue aesthetics in adults	156 ortho-treated vs. 155 non-treated patients; clinical and radiographic assessments; statistical analysis	Orthodontic treatment shows minimal negative effects. Significant differences in tooth display, gingival width, and crestal bone level.
Azaripour A. et al., 2015 [73]	Cross-sectional study	100 patients: 50 fixed orthodontic appliance (FOA): 16 male, 34 female; 50 Invisalign: 11 male, 39 female	50	50	Clinical examinations, questionnaires, statistical analysis using Mann–Whitney U-test and Fisher’s exact test	To compare oral health status, oral hygiene, and patient satisfaction between FOA and Invisalign.	Invisalign patients showed better periodontal health, lower gingival index (GI), sulcus bleeding index (SBI), and higher satisfaction than FOA patients.
Hye-Young S. et al., 2017 [74]	Cross-sectional study	14,693 adults (≥19 years)	Not specified	Not specified	Data from the Fifth and Sixth Korean National Health and Nutrition Examination Surveys (KNHANES V, VI-1, VI-2)	To investigate the association between orthodontic treatment and periodontitis in South Korea	History of orthodontic treatment is associated with a lower prevalence of periodontitis; odds ratios: 0.553, 0.614, 0.624 (*p* < 0.0001).
Kumar V. et al., 2021 [75]	Observational study	120 patients: 48 with extractions, 72 without	Not specified	120 (all treated with fixed appliances)	To assess the impact of fixed orthodontic treatment on gingival health	Full intraoral and extraoral examination, radiographs, photographic records, plaque, inflammation, and gingival recession measurements	Significant increase in visible plaque, inflammation, and gingival recession post-treatment. Regular oral prophylaxis is essential during orthodontic treatment.
Levrini L. et al., 2018 [76]	Comparative study	40 patients: 20 males, 20 females	20	20	Analysis of periodontal parameters: plaque index (PI), PD, BOP at baseline (T0), after 1 month (T1), and after 3 months (T2) for both Invisalign and fixed appliances; microbial biofilm measured via polymerase chain reaction (PCR)	To evaluate and compare short-term periodontal effects of Invisalign aligners versus fixed orthodontic appliances	Invisalign aligners resulted in significantly better periodontal health indicators, with lower plaque, BOP, and microbial biofilm, indicating they are a better option for patients with periodontal concerns.
Pango Madariaga A.C. et al., 2020 [77]	Prospective clinical study	40 patients: 20 fixed group (FG), 20 clear aligners group (CAG); FG: mean age 20.6 ± 8.1 years, CAG: mean age 34.7 ± 12.5 years	20	20	Measurements of PD, PI, BOP, and gingival recession (REC) at baseline (T0) and after 3 months (T1); individualized tooth brushing technique; bi-weekly follow-ups	To evaluate periodontal health in orthodontic patients undergoing different treatments	Significant improvements in PD, BOP, and PI in both groups; no significant differences in periodontal health between fixed appliances and clear aligners
Ravera S. et al., 2024 [78]	Prospective pilot study	21 patients: Stage IV periodontitis, gender not specified	21	0	Analysis of periodontal metrics: PPD, REC, CAL at T0, T1, T2; intraoral scans using iTero Element 5D; clear aligner treatment (Invisalign)	To analyze periodontal response to clear aligner therapy in patients with severe periodontitis	Significant reduction in PPD and CAL; slight increase in REC; digital data showed decreased gingival recession and clinical crown length; clear aligners effective for severe periodontitis

**Table 3:** Risk of bias of the articles.

Authors	D1	D2	D3	D4	D5	D6	D7	Overall
Abbate G.M. et al., 2015 [71]	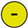	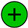	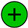	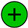	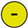	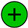	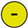	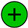
Abdelhafez R.S. et al., 2021 [72]	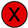	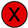	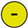	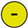	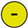	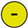	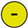	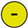
Azaripour A. et al., 2015 [73]	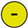	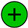	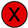	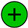	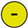	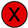	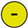	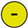
Hye-Young S. et al., 2017 [74]	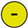	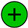	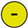	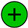	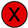	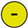	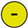	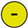
Kumar V. et al., 2021 [75]	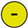	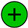	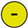	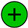	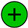	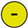	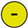	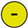
Levrini L. et al., 2018 [76]	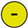	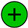	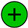	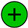	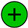	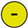	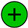	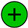
Pango Madariaga A.C. et al., 2020 [77]	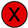	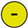	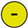	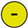	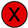	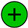	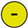	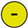
Ravera S. et al., 2024 [78]	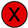	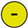	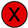	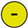	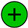	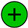	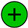	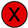
Domains: D1: Bias due to confounding D2: Bias arising from the measurement of exposure D3: Bias in the selection of participants in the study (or in the analysis) D4: Bias due to post-exposure interventions D5: Bias due to missing data D6: Bias arising from the measurement of the outcome D7: Bias in selection of the reported result					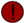 Very High 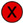 High 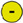 Some Concerns 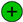 Low 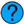 No information			

## Data Availability

The data are contained within the article.
